# Predictive and Prognostic Significance of Molecular Biomarkers in Glioblastoma

**DOI:** 10.3390/biomedicines12122664

**Published:** 2024-11-22

**Authors:** Siddharth Shah, Aiswarya Nag, Sirpi Vivekanandam Sachithanandam, Brandon Lucke-Wold

**Affiliations:** Department of Neurosurgery, University of Florida, Gainesville, FL 32608, USA; aish.nag97@gmail.com (A.N.); sirpi12th@gmail.com (S.V.S.)

**Keywords:** molecular biology, glioblastoma, CNS tumor, diagnostic, prognosis, outcomes

## Abstract

Glioblastoma multiforme (GBM), a WHO grade 4 glioma, is the most common and aggressive primary brain tumor, characterized by rapid progression and poor prognosis. The heterogeneity of GBM complicates diagnosis and treatment, driving research into molecular biomarkers that can offer insights into tumor behavior and guide personalized therapies. This review explores recent advances in molecular biomarkers, highlighting their potential to improve diagnosis and treatment outcomes in GBM patients. Key biomarkers such as MGMT promoter methylation, IDH1/2 mutations, EGFR amplification, and TERT promoter mutations, etc., are examined for their roles in prognosis, therapeutic response, and tumor classification. While molecular biomarkers offer valuable insights for tailoring GBM treatments, their clinical application is hindered by tumor heterogeneity, dynamic genetic evolution, and the lack of standardized testing methods. Future research should aim to confirm new biomarkers and incorporate them into regular clinical practice to improve prognosis and treatment choices. Advances in genomic and proteomic technologies, along with consistent biomarker detection, could transform GBM care and enhance patient outcomes.

## 1. Introduction

Glioblastoma multiforme (GBM), a WHO grade 4 glioma, is the most common malignant primary brain tumor. About 90% develop de novo in older patients (known as primary glioblastomas), while secondary GBMs progress from lower-grade astrocytomas, primarily affecting younger individuals [1,2]. Recent advancements in GBM treatment have been remarkable, particularly in immunology, molecular biology, and virotherapy [1]. Advancements in genomics and proteomics have led to the discovery of numerous molecular biomarkers, drawing significant research attention. These biomarkers, obtained through biopsy or fluid samples, are analyzed using polymerase chain reaction (PCR) and computer-based tools [3].

**Figure 1:**
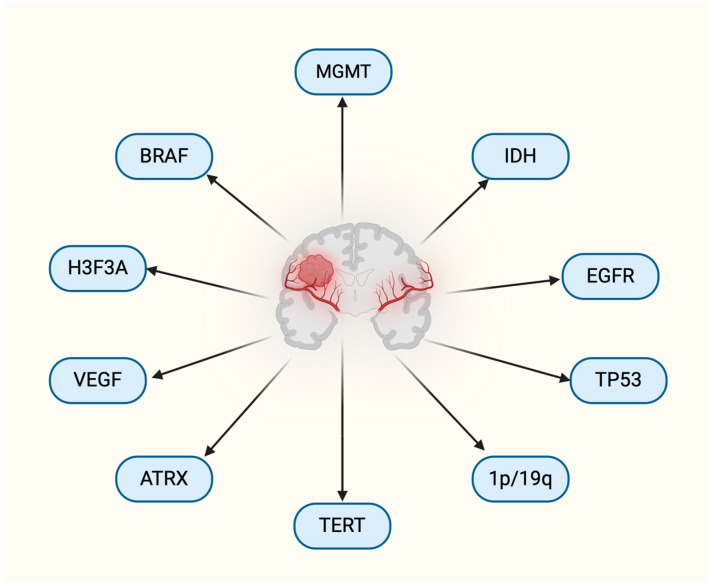
Outline of important biomarkers for GBM.

The poor prognosis of GBM is primarily due to late diagnosis, diffuse infiltration, necrosis, microvascular proliferation, and resistance to treatment, all exacerbated by significant tumor heterogeneity and the adaptable nature of GBM cells, which are influenced by a local inflammatory microenvironment that promotes aggressiveness and drug resistance [4]. Liquid biopsy is a non-invasive promising technique of cancer diagnosis by enabling real-time detection and monitoring of central nervous system (CNS) tumors, potentially replacing traditional tissue biopsies. It analyzes components like circulating tumor cells (CTCs), circulating tumor DNAs (ctDNA), tumor microRNA (miRNAs), exosomes and other metabolites from cerebrospinal fluid (CSF), plasma, and serum, which cross the blood–brain barrier, thus revealing tumor heterogeneity and improving prognosis [5,6]. Liquid biopsy-based circulating biomarkers have potential in GBM diagnostics but are limited by methodological inconsistencies; they should complement tissue biopsies and utilize diverse biomarkers to enhance accuracy and address existing challenges [6]. Mutations critical for GBM classification play a significant role in tumorigenesis, with key genes such as O6 methylguanine methyltransferase (MGMT), isocitrate dehydrogenase (IDH1/2), p53, epidermal growth factor receptor (EGFR), PDGFR, PTEN, PI3K, 1p/19q chromosome deletion, and few others serving as biomarkers of disease aggressiveness and potential targets for treatment [7]. MGMT and IDH1 are established biomarkers associated with patient outcomes, and recent studies reaffirm that MGMT promoter methylation significantly correlates with improved overall survival (OS) in GBM patients, regardless of treatment. Although MGMT is traditionally linked to alkylating agents, its methylation appears beneficial across various therapies. In contrast, the role of EGFR frequently altered in GBM through mutations, amplifications, and the formation of EGFRvIII, remains unclear, with conflicting evidence on its prognostic value. Additional biomarkers like circulating markers may enhance treatment management in future clinical trials [8,9]. Figure 1 provides an outline of biomarkers for GBM.

Despite advances in GBM research, patient outcomes remain poor, with diagnosis still relying heavily on invasive tissue biopsies [9]. Prognostic and predictive biomarkers are crucial in gliomas, helping to assess prognosis, guide therapy, and differentiate pseudoprogression from true tumor progression, with new biomarkers emerging from advances in sequencing technologies to improve patient management [10]. In the future, biomarkers, individually or in combination, are expected to more accurately identify the pathogenic GBM subtype and guide therapy selection [11]. This review delves into the latest developments in GBM biomarkers and their capacity to enhance diagnosis, prognosis, and treatment choices in clinical settings.

## 2. Molecular Picture of Glioblastoma

Primary GBM has a poorer prognosis than secondary GBM, often exhibiting EGFR overexpression, 1p/10q LOH, and TERT promoter mutation and PTEN mutations, while secondary GBM is typically associated with IDH mutation, ATRX mutations, and TP53 mutations [3,8]. Abnormal gene activity and changes in gene copy numbers have helped identify four types of GBM (glioblastoma) based on the Verhaak classification: classical, mesenchymal, proneural, and neural [11,12]. Glioblastomas, primarily IDH-wild-type tumors, are challenging to treat due to the lack of known risk factors and ineffective pharmacological interventions; however, specific biomarkers like BRAF mutations and gene fusions (e.g., NTRK, FGFR) present potential targeted therapy opportunities [13]. EGFR and PTEN are crucial components of the receptor tyrosine kinase (RTK) pathway, frequently altered in primary GBM. PTEN inhibits the RTK pathway and is mutated in 15–40% of primary GBMs. GBM’s reliance on angiogenesis for growth is mainly regulated by vascular endothelial growth factor (VEGF), produced in response to tumor hypoxia and by GBM cancer stem cells [14].

The use of histopathology and sequencing to assess GBMs is limited due to their invasiveness and failure to consider tumor diversity, suggesting that imaging biomarkers could improve monitoring. As immunotherapy progresses, the need for effective biomarkers will increase, aiding in better treatment decisions and outcomes while minimizing ineffective therapies [15,16,17]. The most advanced biomarkers are immune checkpoint inhibitors. MicroRNAs like miR-10b and miR-21 serve as prognostic biomarkers and are crucial in developing anti-GBM therapies, including vaccines, antibody-drug conjugates, and growth factor receptor inhibitors [18,19]. GBM’s internal heterogeneity complicates diagnosis and prognosis, as tumor cells within the same mass can vary spatially and temporally. This makes it difficult to validate biomarkers and treat, as tumor growth and drug resistance are influenced by interactions with the surrounding microenvironment, altered extracellular matrix, and pathological metabolism [20,21]. GBM pathogenesis involves complex genetic, epigenetic, and transcriptomic alterations that ultimately disrupt key signaling pathways [22]. Different tumor subtypes require personalized approaches. Biomarker profiles such as IDH1, EGFR, and MGMT, along with age-specific mutations in genes like TP53 and ATRX, highlight the need for customized therapies based on genetic variations and tumor behavior [23,24]. Near-complete tumor resection is also linked to a better prognosis in patients with GBM [25].

**Figure 2:**
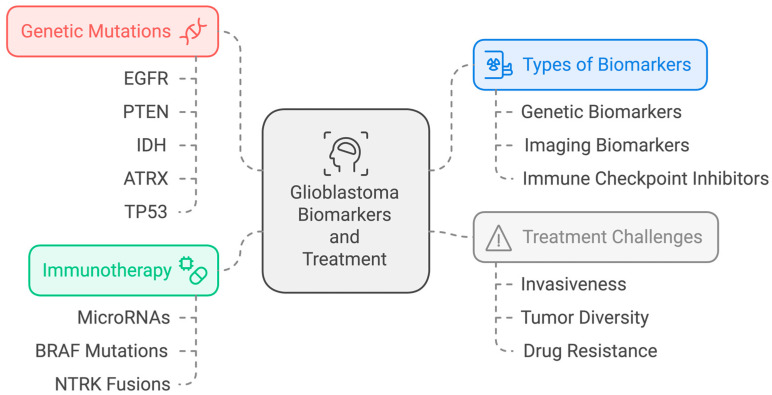
Summary of the GBM biomarkers and treatment options.

An ideal tumor marker for GBM should be easily accessible, highly sensitive and specific, and provide accurate information on the disease’s presence and severity. However, most GBM biomarkers lack either sensitivity or specificity. Genetic profiling, such as IDH1/2 mutation and MGMT promoter methylation, is now crucial for diagnosis and prognosis. Despite advancements in nucleic acid analysis techniques, the detection of tumor-derived nucleic acids in peripheral blood remains challenging due to low concentration and weak penetration across the blood–brain barrier [20]. Figure 2 summarizes the biomarkers and treatment options for GBM. Table 1 provides a summary of the important molecular biomarkers in GBM.

## 3. Overview of Molecular Biomarkers

### 3.1. MGMT Promoter Methylation

MGMT promoter methylation serves as a positive predictive biomarker for patients treated with alkylating agents like temozolomide (TMZ) chemotherapy. By reducing the expression of the MGMT gene, which encodes a DNA repair protein, this methylation makes cells more susceptible to the cytotoxic cell death in tumor cells by alkylating agents [8,26]. Studies indicate that low MGMT protein or gene expression is significantly linked to improved survival and treatment response, serving as an independent prognostic marker and predictor of response to alkylating therapies in GBM patients [27,28]. The predictive biomarker status of MGMT methylation in prognosis may be influenced by age, gender, and race [29]. The MGMT gene is located on chromosome 10 (10q26). In most GBMs, one allele of this chromosome is frequently lost, leaving the remaining copy to drive gene function [30]. MGMT is a suicide DNA repair protein that transfers a methyl group from the O6-position of guanine to its own cysteine residue, which becomes crucial when treating gliomas with alkylating agents like tTMZ. In hypermethylated MGMT tumors, reduced MGMT expression enhances the cytotoxic effects of alkylating drugs, increasing treatment efficacy [31]. MGMT expression is regulated by various mechanisms, including promoter methylation, histone modifications, transcription factors, and microRNA interactions, which influence its role in DNA repair and resistance to alkylating agents like TMZ [32,33]. Combining analyses of methylation and expression yields more accurate predictions of patient outcomes. For example, patients with both MGMT methylation and low protein expression show the longest survival, while unmethylated–immunonegative patients have the poorest prognosis [28].

A study comparing MGMT promoter methylation detection found that CSF samples exhibited higher sensitivity than serum samples, suggesting that CSF analysis could serve as a minimally invasive alternative for diagnosis and monitoring in patients unable to undergo traditional tissue sampling [34]. Treatment decisions for elderly GBM patients should take MGMT status into account, and it is reasonable to withhold TMZ from those with newly diagnosed GBM lacking MGMT promoter methylation in clinical trial settings [29]. MGMT promoter methylation plays a crucial dual role in managing glioblastoma, both as a predictor of patient sensitivity to TMZ and as a marker of overall prognosis. MGMT methylation silences the gene responsible for repairing TMZ-induced DNA damage, making methylated tumors more responsive to this chemotherapy and resulting in improved survival outcomes for these patients [30]. However, this biomarker’s value is complicated by evidence that MGMT methylation status can change throughout treatment. Recent studies suggest that methylation patterns may shift in response to the selective pressures of therapy, potentially contributing to treatment resistance as unmethylated tumor subpopulations expand over time [31]. Understanding the dynamic nature of MGMT methylation could inform treatment adaptations, such as adjusting therapy based on changes in methylation status, to maintain treatment efficacy and potentially improve patient outcomes [34]. Addressing this evolving characteristic may help develop more personalized approaches that consider both initial and adaptive tumor biology in GBM care.

Patients with MGMT promoter methylation in GBM generally have better survival outcomes compared to those without this methylation. MGMT promoter methylation silences the MGMT gene, which normally repairs DNA damage. When this gene is methylated, tumors are less able to repair the damage caused by alkylating chemotherapy agents like TMZ, making the cancer cells more vulnerable to treatment [28]. Studies indicate that patients with MGMT promoter-methylated GBM experience prolonged progression-free survival (PFS) and overall survival (OS), particularly when treated with TMZ, as their tumors are more responsive to this chemotherapy. For instance, some data suggest that MGMT-methylated patients may have median overall survival rates of up to 21–23 months, whereas those without methylation often experience median survival around 12–15 months [2]. Consequently, MGMT promoter methylation has become an important prognostic biomarker, helping clinicians tailor treatment approaches and predict outcomes more accurately for GBM patients.

### 3.2. EGFR Amplification

Epidermal growth factor receptor (EGFR), also known as HER1 or ERBB1, a transmembrane receptor tyrosine kinase (RTK), is commonly altered in GBM through mutations, rearrangements, splicing, and amplifications, playing a key role in cell division, migration, and apoptosis [10,35]. In glioblastoma, EGFR gene amplification is the most common RTK mutation, occurring in approximately 40% of cases, predominantly in primary GBM. While early studies linked EGFR amplification to poor prognosis, subsequent research has not consistently supported this, and EGFR amplifications have not proven effective as independent predictive biomarkers in clinical trials for EGFR inhibitors [36]. Significant correlations between EGFR expression and immune cell infiltrates, including B cells, CD8+ T cells, and macrophages, was found to indicate their potential as prognostic and therapeutic biomarkers. Despite no statistical link between EGFR expression and GBM outcomes, high EGFR expression was still associated with a poor prognosis in GBM patients [37]. EGFR amplification, especially with EGFRvIII mutation, is often linked to poor prognosis in GBM, though some studies show better outcomes in specific groups, like those with high amplification or CDKN2A deletion. EGFRvIII’s prognostic value is inconsistent, with mixed evidence on its impact on survival. Overall, the role of EGFR amplification and mutation in predicting prognosis remains unclear [38]. Further biomarker-enriched clinical studies could provide deeper insights into the potential benefits of EGFR-targeted therapies [39].

EGFR (epidermal growth factor receptor) amplification is common in GBM and is generally associated with a poorer prognosis. EGFR amplification leads to increased receptor expression and activation, promoting cell proliferation, survival, and tumor invasiveness. Patients with EGFR-amplified GBM tend to experience more aggressive disease progression and shorter overall survival (OS) compared to those without this amplification [38]. Studies suggest that the median OS for patients with EGFR amplification in GBM is often around 12–15 months, which is shorter than for some other molecular subtypes [2]. Although EGFR amplification is linked to poor survival, it also presents a potential therapeutic target. Various clinical trials are evaluating targeted therapies, such as EGFR inhibitors and EGFR-specific CAR-T cell therapies, to assess their efficacy in EGFR-amplified glioblastoma. However, these treatments have shown limited success so far, largely due to tumor heterogeneity and the development of resistance mechanisms. This ongoing research highlights the potential and challenges of leveraging EGFR amplification both as a prognostic marker and as a target for new treatment approaches [39].

### 3.3. IDH 1/2 Mutations

Isocitrate dehydrogenases (IDH1, IDH2, and IDH3) facilitate the conversion of isocitrate to alpha-ketoglutarate while reducing NADP to NADPH. This process is essential for regenerating reduced glutathione, the primary antioxidant in cells. Mutations in IDH1, primarily the R132H single amino acid substitution, result in the loss of normal enzyme function and are mostly heterozygous [40]. In a study of patients with GBM who did not have IDH mutations, the median overall survival was about 13 months, and the histopathological features of necrosis and vascular proliferation lost their prognostic significance. In contrast, patients with GBM and IDH mutations showed a significantly better median overall survival of 54 months, highlighting the importance of IDH status in prognosis [41]. IDH1 and IDH2 mutations drive cancer through excessive D-2-hydroxyglutarate (D-2HG) production, disrupting metabolism and epigenetic regulation, and serve as biomarkers for detection and targeted therapy in specific cancers [42]. IDH mutations are more common in secondary glioblastomas and the unique histological features such as microvascular proliferation and necrosis are critical for prognosis, as IDH-mutant GBMs have shorter recurrence-free and overall survival. This also aids in distinguishing between the two types of GBM [43,44]. Most IDH1-mutant GBMs (92%) exhibit a proneural expression signature, indicating that secondary glioblastomas are relatively homogeneous, while primary glioblastomas are more heterogeneous with various expression profiles [2]. A study highlighted the role of IDH1/IDH2 mutations in GBMs, as they convert alpha-ketoglutarate to the oncometabolite 2-hydroxyglutarate, crucial for gliomagenesis [45]. IDH has been proven as a reliable prognostic marker by several studies, but a two-gene predictor study by Molenaar et al. correlates with progression-free survival linked to the genetic and epigenetic status of IDH1 and/or MGMT in both primary and secondary glioblastomas [46,47]. GBMs with mutated IDH1 and associated epigenetic changes tend to have a longer PFS as well as OS and could be used as a strong prognostic marker; however, they are not independent prognostic factors, underscoring the necessity for further research to elucidate their role and improve their utility as treatment biomarkers [40,48,49,50].

IDH1 and IDH2 mutations in GBM are associated with a significantly better prognosis and longer survival compared to patients with wild-type (non-mutated) IDH. These mutations are more commonly found in lower-grade gliomas and secondary glioblastomas, which tend to progress more slowly and respond better to treatment than primary (de novo) glioblastomas [48]. Studies show that patients with IDH-mutated glioblastomas often have a median overall survival (OS) of 24–36 months, or even longer in some cases, compared to a typical median OS of 12–15 months for those with IDH wild-type tumors [2]. The presence of an IDH mutation is thought to alter tumor metabolism in a way that limits tumor growth, and it has been linked to improved responses to chemotherapy and radiotherapy. IDH-mutant gliomas are also often associated with other favorable biomarkers, such as ATRX mutations and 1p/19q codeletions, further contributing to better outcomes [49]. Due to these survival benefits, IDH1/2 mutations are not only valuable prognostic biomarkers but are also being investigated as potential therapeutic targets, with IDH inhibitors currently in clinical trials aiming to improve survival for this subset of glioma patients [50].

### 3.4. Loss of Heterogeneity on Chromosome

Loss of heterozygosity (LOH) on chromosome 19q plays a significant role in the progression from low-grade astrocytomas to secondary glioblastomas, but not in primary glioblastomas [51]. Codeletion of 1p/19q is associated with improved overall survival, particularly when combined with IDH mutations and other favorable genomic alterations, while its presence is negatively correlated with p53 expression [52]. Codeletion of 1p/19q is linked to improved progression-free and overall survival rates, with isodeletion of 1p also indicating favorable outcomes, while the impact of isodeletion of 19q is marginal [53]. LOH on chromosome 10 is common in both primary and secondary GBM, with primary GBM generally losing the entire chromosome 10, suggesting the presence of multiple tumor-suppressor genes on this chromosome that may play a role in GBM tumorigenesis. Secondary GBMs mainly lose 10q but retain 10p, highlighting distinct genetic mechanisms in their development [54,55]. GBM’s pronounced intratumoral heterogeneity—where different regions or cell populations within a single tumor exhibit distinct molecular signatures—poses a significant challenge for effective treatment. This heterogeneity often results in varying responses to therapy across different tumor subtypes, as cells with particular mutations or alterations may evade treatment, leading to resistance and disease progression [55]. For example, while one tumor region may show sensitivity to a specific therapy targeting EGFR amplification, another region might harbor mutations like TERT promoter alterations that drive treatment resistance. This molecular diversity not only complicates targeted therapies but also underscores the need for multi-targeted approaches that can simultaneously address the tumor’s various subpopulations. Understanding and characterizing these distinct molecular landscapes within GBM are critical steps toward developing therapies that can adapt to, or overcome, this resistance [54]. By mapping out these variations, researchers could design more comprehensive treatment strategies that better manage the complexity of GBM and potentially improve patient outcomes.

### 3.5. TERT Promoter Mutations

Telomerase reverse transcriptase (TERT) promoter mutations C228T and C250T are critical to malignant transformation in GBM. By activating telomerase, they drive oncogenesis, enabling cellular immortalization, making them significant biomarkers [56]. The 2021 WHO Classification of Primary CNS Tumors identifies TERT promoter mutations as crucial diagnostic markers for CNS tumors, particularly in oligodendroglioma, glioblastoma, and meningioma, with a high mutation frequency of 70% in glioblastomas [57]. Telomere maintenance mechanisms are crucial during DNA replication in glioblastomas, with TERT promoter mutations being the most prevalent alterations, indicating their key role in oncogenesis and integration into current GBM diagnostic procedures [58]. TERT mutations are common, especially in primary GBMs, and are linked to poorer overall survival. A study by Simon et al. found that TERT mutations identify different subgroups of GBMs with varying levels of malignancy and may help guide treatment strategies [59,60]. In a GBM study, human TERT mutations (hTERT) were found in 75% of patients but did not predict survival alone; however, when combined with MGMT methylation, those with hTERT mutations had significantly better survival, suggesting the effect of MGMT on prognosis depends on hTERT status [61]. In a different study, patients with only TERT mutations showed the shortest survival, while those with both TERT and IDH mutations had the longest survival, highlighting the significance of telomere biology in disease outcomes [62].

TERT promoter mutations are common in GBM and are generally associated with a poorer prognosis. These mutations lead to increased telomerase activity, allowing cancer cells to maintain their telomeres and continue dividing, which contributes to tumor growth and aggressiveness [61]. Patients with TERT promoter-mutated glioblastomas often experience shorter overall survival (OS) compared to those without the mutation. For instance, studies have shown that the median OS for patients with TERT promoter mutations can range from 12 to 16 months, which is lower than for some other molecular subtypes of GBM [2]. The prognostic impact of TERT promoter mutations is even more pronounced when combined with other genetic markers. For example, glioblastoma patients with both TERT promoter mutations and EGFR amplification or wild-type IDH often have a worse prognosis compared to those with tumors with only one of these alterations [63]. However, in tumors where TERT promoter mutations coexist with IDH mutations, as is more commonly seen in lower-grade gliomas, patients may experience relatively better outcomes. [63] Understanding TERT promoter mutations is therefore valuable for refining prognosis and may also guide therapeutic strategies as research continues to explore telomerase inhibition as a potential treatment approach for glioblastoma.

### 3.6. ATRX Mutations

The ATRX gene, found on chromosome Xq21.1, encodes a protein involved in the H3.3-ATRX-DAXX chromatin-remodeling pathway [63]. ATRX alterations frequently co-occur with other mutations (such as IDH1 and TP53) and are associated with improved overall survival, highlighting its potential as a diagnostic and therapeutic target in GBM [64]. ATRX gene mutations lead to the loss of nuclear protein expression in tumor cells while preserving expression in non-tumor cells like endothelial and pre-existing glial cells, which act as a positive internal control [63]. Patients with ATRX loss had longer survival and time before disease progression compared to those without ATRX loss, but the difference was not statistically significant [65]. ATRX mutations are common in secondary glioblastomas, particularly in IDH-mutant glioblastoma, but are rare in IDH wild-type patients, who generally have better survival outcomes [9]. A study showed that the absence of ATRX promotes sister telomere recombination and genomic instability. ATRX mutations are linked to younger patients and improved survival in GBM, best detected by immunohistochemistry (IHC) [66].

ATRX mutations in GBM are generally associated with a more favorable prognosis, particularly in tumors that also harbor IDH mutations. ATRX (alpha-thalassemia/mental retardation syndrome X-linked) mutations are often found in secondary glioblastomas and are typically linked with alternative lengthening of telomeres (ALT), a telomerase-independent mechanism for maintaining telomere length [64]. Patients with ATRX-mutated gliomas, especially in the context of concurrent IDH mutations, tend to have longer survival outcomes compared to patients with ATRX-wild-type glioblastomas [65]. Studies indicate that the median overall survival (OS) for patients with ATRX mutations in the context of IDH-mutant gliomas can extend beyond 24–36 months, significantly longer than the 12–15 months commonly observed in primary (IDH wild-type) glioblastomas [2]. ATRX mutations are also associated with other favorable prognostic markers, such as 1p/19q codeletions in lower-grade gliomas, further contributing to better outcomes in this subgroup [66]. While ATRX mutations themselves are not currently direct therapeutic targets, their presence is valuable for prognostication and can help refine treatment approaches, especially when considering the overall molecular profile of the tumor.

### 3.7. TP53 Mutations

The TP53 gene, located on chromosome 17q13.1, encodes the p53 protein, a key tumor suppressor and transcription factor involved in regulating cell growth and preventing cancer, including glioblastoma. TP53 is crucial for cell cycle regulation and apoptosis, frequently mutated or deleted in glioblastomas, with alterations often coexisting with IDH mutations, promoting glioma progression. TP53 mutations, seen in primary and secondary glioblastomas, are linked to poor prognosis, though therapies targeting p53, such as gene therapy, have shown some potential [9,66]. The poor prognosis of TP53 is due to increased malignancy, enhanced proliferation, invasion, and chemotherapy resistance [66].

TP53 mutations in GBM are associated with varied prognostic outcomes, as their impact on survival can depend on other co-existing molecular alterations within the tumor. TP53, a tumor suppressor gene, plays a critical role in regulating cell division and apoptosis [66]. Mutations in TP53 are common in secondary glioblastomas and tend to co-occur with IDH1/2 mutations, which are generally linked to better survival outcomes. In IDH-mutant gliomas with TP53 mutations, patients often experience longer median overall survival (OS), sometimes extending beyond 24–36 months, as these tumors tend to grow more slowly [2]. In contrast, TP53 mutations in primary (IDH wild-type) glioblastomas, which are generally more aggressive, are often linked to shorter survival times. Studies show that patients with TP53 mutations in IDH wild-type glioblastomas may have a median OS closer to 12–15 months, which aligns with the typical prognosis for primary GBM. Overall, while TP53 mutations alone are not strongly predictive of survival in GBM, their combined effect with other mutations, such as IDH1/2, can offer valuable prognostic insights. TP53 mutation status is particularly useful when interpreted alongside the tumor’s broader molecular profile, which can help clinicians better understand disease progression and potentially inform treatment approaches.

### 3.8. VEGF Expression

Vascular endothelial growth factor (VEGF), particularly VEGF-A, is a key driver of angiogenesis and tumor progression in glioblastomas. VEGF overexpression, detected in majority of glioblastomas, led to the development of the anti-angiogenic drug Bevacizumab, which blocks tumor vascularization by neutralizing VEGF-A overexpression. The VEGF ligand family includes ligands like VEGF-C, which is overexpressed in GBM and linked to tumor progression, with high levels being a poor prognostic factor [9]. While VEGF is the main angiogenic factor, GBM tumors often also express various other proangiogenic factors, including platelet-derived growth factor (PDGF), fibroblast growth factor (FGF), integrins, hepatocyte growth factor/scatter factor, angiopoietins, ephrins, and interleukin-8 [67]. The plasma level of the VEGF-121 isoform serves as a biomarker for GBM tumors and may help predict responses to anti-angiogenic treatment. However, the predictive value of baseline VEGF-121 levels and the reduction of this isoform after bevacizumab infusion require validation through larger, multicenter clinical studies [68].

VEGF expression in GBM is generally associated with a poorer prognosis, as VEGF promotes angiogenesis, enabling the tumor to form new blood vessels that support rapid growth and invasive behavior. High levels of VEGF are commonly observed in glioblastoma, correlating with aggressive disease features, treatment resistance, and reduced overall survival (OS) [2]. Studies have indicated that GBM patients with elevated VEGF expression often experience shorter median OS, typically around 12–15 months, which aligns with the prognosis for more aggressive GBM cases [67]. VEGF-targeted therapies, such as bevacizumab (an anti-VEGF monoclonal antibody), have been tested in clinical trials to assess whether blocking VEGF can improve survival in GBM patients. While bevacizumab has been shown to reduce tumor-associated edema and improve progression-free survival (PFS), it has not consistently demonstrated a significant benefit in extending overall survival [68]. Thus, while VEGF expression serves as a useful prognostic marker and indicates potential responsiveness to anti-angiogenic therapies, its role in improving survival outcomes remains limited, with ongoing research exploring combination approaches to enhance its therapeutic impact in glioblastoma.

### 3.9. H3F3A and BRAF Mutations

Pediatric GBMs are aggressive tumors, with K27M mutations in the H3F3A gene—encoding histone H3.3—found in approximately 30% of cases and 80% of diffuse intrinsic pontine gliomas [69]. H3F3A mutations impacting K27 and G34 amino acids occur in one-third of pediatric malignant gliomas and are linked to significantly worse outcomes, making the assessment of these protein defects crucial for clinical management in pediatric patients [10]. Another key mutation in young adults is BRAF. BRAF-V600E is indicated as a favorable prognosis, while H3F3A-K27M was linked to poor outcomes, emphasizing the importance of these biomarkers in clinical classification [70].

**Figure 3:**
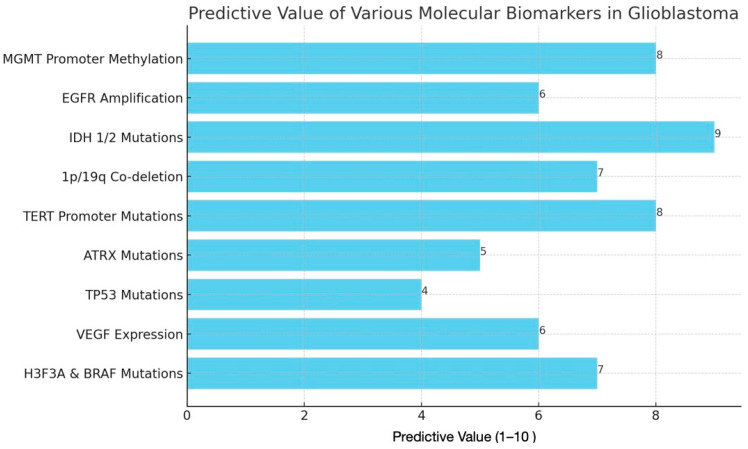
Graphical illustration of the predictive values of molecular biomarkers in GBM. Each biomarker is rated from 1–10, 10 being highly predictive.

Emerging biomarkers like ATRX, H3F3A, and microRNA signatures are gaining attention in GBM research for their potential to improve diagnosis and guide treatment. ATRX mutations, for instance, are often associated with alternative lengthening of telomeres, which contribute to tumor growth and may correlate with specific tumor subtypes and patient outcomes [70]. Similarly, mutations in the H3F3A gene, particularly those affecting histone H3, are linked to more aggressive glioma forms and can help refine prognosis by indicating higher-grade or pediatric GBM variants. In addition to genetic mutations, microRNA (miRNA) signatures are showing promise as non-invasive biomarkers detectable in blood or cerebrospinal fluid, offering insights into tumor behavior and potential resistance mechanisms [69]. These miRNAs can regulate gene expression in pathways critical for GBM progression, making them candidates for targeted therapies. Together, these emerging biomarkers hold promise not only for improving early diagnosis and stratifying patients but also for tailoring therapies to the unique molecular profiles of individual GBM tumors, paving the way for more effective and personalized treatment approaches. Figure 3 represents the predictive value of biomarkers in GBM.

MGMT promoter methylation is a key prognostic biomarker associated with improved survival, since patients with methylated MGMT tend to respond better to TMZ chemotherapy. IDH1 and IDH2 mutations are also associated with a better prognosis and are often found in lower-grade gliomas and secondary GBMs, which generally progress more slowly than primary GBMs. Other markers, such as EGFR amplification and TERT promoter mutations, are linked to more aggressive tumor behavior and shorter survival times. By identifying these and other molecular features, clinicians can better predict disease trajectory, enabling more personalized treatment planning and setting more accurate expectations for patient outcomes. Prognostic biomarkers not only help guide initial treatment decisions but also provide valuable information for monitoring disease progression and adjusting therapeutic strategies as needed.

## 4. Clinical Impact of Biomarkers

Molecular biomarkers in GBM hold significant potential for guiding personalized treatment, yet their clinical implications remain complex. Biomarkers like MGMT promoter methylation are predictive of the response to temozolomide chemotherapy, enhancing therapeutic decision-making [71,72]. IDH1 mutations are associated with better prognosis and are commonly used in stratifying GBM subtypes, offering prognostic value [73,74]. However, the heterogeneity of GBM limits the efficacy of single-site biopsies, and the tumor’s genetic evolution over time reduces the predictive reliability of early-stage biomarkers [75]. GBM tumor heterogeneity arises from clonal evolution, cancer stem cells, and interclonal interactions, leading to varied treatment responses and challenges in effective patient management [76]. Moreover, the absence of standardization in testing platforms, such as for EGFR or PDGFRA amplification, complicates consistent clinical application [77,78]. While biomarkers can help direct treatment, their ability to significantly improve overall survival in GBM patients is limited because there are few effective treatment options available [78]. Thus, while biomarkers hold promise, their role in clinical decision-making for GBM requires further refinement and validation [79].

## 5. Conclusions

In conclusion, molecular indicators shed important light on the treatment response and prognosis of glioblastoma, one of the most aggressive types of brain cancer. Well-established biomarkers, including IDH1 mutations and MGMT promoter methylation, have previously been shown to be important in adjusting therapy for specific patients, providing a more individualized level of care. Though these biomarkers show potential, a number of obstacles prevent them from being fully utilized in clinical settings. The trustworthiness of biomarker data is complicated by tumor heterogeneity, wherein unique genetic features may be exhibited in different parts of the same tumor. Furthermore, the genetic makeup of GBM changes over time, which reduces the usefulness of static biomarkers even further. Another obstacle that lowers the repeatability and accuracy of biomarker-driven research is inconsistent testing procedures between clinical settings. Continued research is crucial to overcoming these constraints. Researchers need to keep looking for and confirming novel biomarkers, since they may provide new avenues for more targeted therapies. The direction of future research should focus on validating and expanding the pool of novel biomarkers, particularly those associated with key therapeutic targets in glioblastoma. This research should prioritize biomarkers that not only predict tumor behavior but also identify patient-specific responses to treatments, which could lead to more tailored, effective therapies. In terms of standardization, it is essential to establish uniform protocols for biomarker testing, encompassing sample collection, processing, and interpretation. This will help ensure that results are consistent and reliable across different clinical settings, ultimately enabling biomarker-based treatment strategies to become routine in GBM care. Additionally, standardization needs to be strengthened in order to include these biomarkers into clinical practice. This will guarantee that testing procedures are dependable and uniform across various laboratories and establishments. It is also essential to increase the number of treatment options that target these molecular alterations precisely. Establishing a therapeutic paradigm that not only makes biomarker-guided care feasible but also greatly enhances patient outcomes is the ultimate objective. By tackling these obstacles, biomarkers’ potential to completely transform the treatment of GBM can be achieved, giving patients with this terrible illness hope for a better prognosis, more efficacious therapies, and increased survival rates.

Expanding on the potential of biomarkers to drive treatment decisions in glioblastoma, several clinical trials and research initiatives are focusing on biomarker-driven therapies that could transform precision oncology for this challenging disease. Personalized vaccines, such as neoantigen-based vaccines, are under investigation to trigger immune responses tailored to the unique mutations in a patient’s tumor, with recent trials showing promise in prolonging survival for select patients with specific biomarker profiles. Targeted therapies are also being explored, including EGFR inhibitors and IDH1/2 inhibitors, which aim to exploit vulnerabilities in tumors with certain genetic alterations. Biomarkers like MGMT methylation, IDH mutations, and EGFR amplifications are increasingly used in trial designs to stratify patients and assess treatment efficacy within specific molecular subgroups. Additionally, CAR-T cell therapies targeting molecular markers unique to GBM cells are in early-stage trials, aiming to harness the immune system to selectively attack tumor cells. By focusing on biomarker-driven strategies, these studies are advancing the field of precision oncology in glioblastoma, with the potential to shift away from conventional, one-size-fits-all treatments toward more effective, individualized therapeutic approaches. This ongoing research brings practical relevance to biomarker identification, highlighting how specific molecular alterations may soon directly inform treatment options, ultimately improving outcomes in this aggressive cancer.

## 6. Limitations of Biomarker Use

Using biomarkers in GBM treatment and prognosis faces several significant obstacles and limitations, which complicate their integration into routine clinical practice. One primary challenge is the high degree of intratumoral heterogeneity, where distinct regions within the same tumor display different genetic and molecular profiles. This variability can make it difficult to capture a comprehensive biomarker profile with a single biopsy and may lead to inaccurate predictions of treatment response. Additionally, GBM’s molecular landscape can evolve over time, especially under therapeutic pressure, leading to shifts in biomarker expression that may contribute to treatment resistance and complicate long-term treatment planning.

Another limitation is the lack of standardized testing protocols for biomarkers. Different diagnostic platforms—such as immunohistochemistry, next-generation sequencing, and methylation profiling—each have unique advantages and limitations but can produce inconsistent results, hindering comparability across clinical settings. This variability often affects the reliability and reproducibility of biomarker-based diagnostics, making it challenging for clinicians to make confident, consistent decisions based on these markers.

Lastly, many GBM biomarkers, though promising, are still being validated and require robust clinical evidence before they can be fully endorsed for widespread use. This often slows down the process of translating biomarker discoveries into practical, approved clinical tools. Consequently, while biomarkers hold great potential to improve GBM care, overcoming these obstacles is essential to unlock their full benefit for personalized, precision-based treatment.

## 7. Limitations and Challenges

GBM tumors are highly heterogeneous at both the genetic and molecular levels, making it difficult for a single biopsy to capture the tumor’s full profile, as different areas may show varying mutations. Despite research, only a few molecular biomarkers (e.g., IDH mutations, MGMT promoter methylation) are reliably predictive or prognostic. Newer biomarkers often lack validation in large-scale clinical trials, making them unreliable in clinical use. The genetic profile of GBM can also evolve during treatment, reducing the relevance of biomarkers tested only at diagnosis. Some molecular biomarkers require advanced, technically challenging methods for detection (e.g., next-generation sequencing or methylation arrays), which may not be widely available, especially in less-resourced settings. Variations in testing platforms can also lead to discrepancies in results. While molecular biomarkers like MGMT methylation status are associated with treatment response (e.g., to temozolomide), their impact on clinical decision-making is still limited, particularly because treatment options for GBM remain constrained and survival outcomes are generally poor despite biomarker-guided treatment. Additionally, many biomarkers are not clearly categorized as prognostic or predictive, a distinction essential for their use in clinical practice.

A critical barrier to the effective use of molecular biomarkers in GBM care is the lack of standardized testing methods, which results in variable results across diagnostic platforms. Techniques such as immunohistochemistry (IHC), next-generation sequencing (NGS), and methylation arrays each have distinct strengths and limitations but lack harmonization, making it challenging to integrate biomarker data consistently. For instance, IHC is relatively cost-effective and widely accessible, yet it may lack the sensitivity to detect low-abundance mutations that NGS can reveal. NGS, while highly sensitive, is costly and resource-intensive, limiting its availability in many clinical settings. Methylation arrays, which can provide comprehensive methylation profiling, are effective for identifying methylation-based biomarkers like MGMT promoter status but may not capture non-methylation-based mutations effectively. Without standardized protocols and cross-platform calibration, results can vary significantly depending on the platform and lab, leading to inconsistent clinical decisions. Harmonizing these diagnostic methods—perhaps by creating a universal testing panel or by setting clear guidelines for combining multiple techniques—would allow clinicians to rely on biomarker data with greater confidence, ultimately advancing biomarker-guided care in glioblastoma.

## Figures and Tables

**Table 1:** Summary of important molecular biomarkers in glioblastoma (neural subtype not used since it is not distinctly represented by the listed biomarker). EGFR: epidermal growth factor receptor; MGMT: O^6^-methylguanine-DNA methyltransferase; 1p/19q codeletion: chromosomal arm 1p and 19q codeletion; TP53: tumor protein 53; IDH1/2: isocitrate dehydrogenase 1/2; TERT: telomerase reverse transcriptase; ATRX: alpha thalassemia/mental retardation syndrome X-linked; PDGFRA: platelet-derived growth factor receptor alpha; H3F3A: H3 histone family member A; PTEN: phosphatase and tensin homolog; VEGF: vascular endothelial growth factor.

Biomarker	GBM Classification	GBM Type (Primary/Secondary)	Clinical Application	Remarks
EGFR	Classical	Primary	Prognostic biomarker	Frequently amplified, playing a central role in promoting tumor growth and survival.
MGMT	Classical	Primary	Predictive and prognostic biomarker	Methylation status may be present, impacting treatment response.
1p/19q codeletion	Classical	Primary	Prognostic biomarker	Signify progression from lower-grade tumors and associated with improved outcomes.
TP53	Classical	Primary and secondary	Prognostic biomarker	Mutations associated with genomic instability and enhancing the invasive characteristics of the tumor.
IDH1/2	Proneural	Secondary	Prognostic biomarker	Mutations are often linked to a better prognosis and distinct tumor biology.
TERT	Proneural	Primary	Diagnostic and prognostic biomarker	Promoter mutations contribute to telomere maintenance and tumor progression, associated with poor prognosis.
ATRX	Proneural	Secondary	Prognostic biomarker	Frequently mutated, with its loss linked to alternative lengthening of telomeres and tumor progression.
PDGFRA	Proneural	Secondary	Prognostic biomarker	Alterations can be present, though less common than in other subtypes.
H3F3A	Proneural	Secondary	Predictive biomarker	Mutations can be found in particularly in pediatric patients.
PTEN	Mesenchymal	Primary	Prognostic biomarker	Loss or mutation is linked to increased tumor aggressiveness and worse outcomes.
VEGF	Mesenchymal	Primary and Secondary	Prognostic biomarker	Overexpressed, contributing to increased angiogenesis and aggressive tumor behavior.

## Data Availability

Not applicable.
